# Supplementary material for: DMRforPairs: identifying Differentially Methylated Regions between unique samples using array based methylation profiles
Source: BMC Bioinformatics. 2014 May 15;15:141. doi: 10.1186/1471-2105-15-141 (PMC4046028; doi:10.1186/1471-2105-15-141)
Supplement: Additional file 2 — DMRforPairs output for the comparison of A431-MCF7 and NA17018-NA17105. Please start from the HTML files in each folder. Available via the BMC Bioinformatics website. [file 1471-2105-15-141-S2.zip › 1394847754114233_MOESM2_ESM/A431_MCF7/figures/31218.pdf]

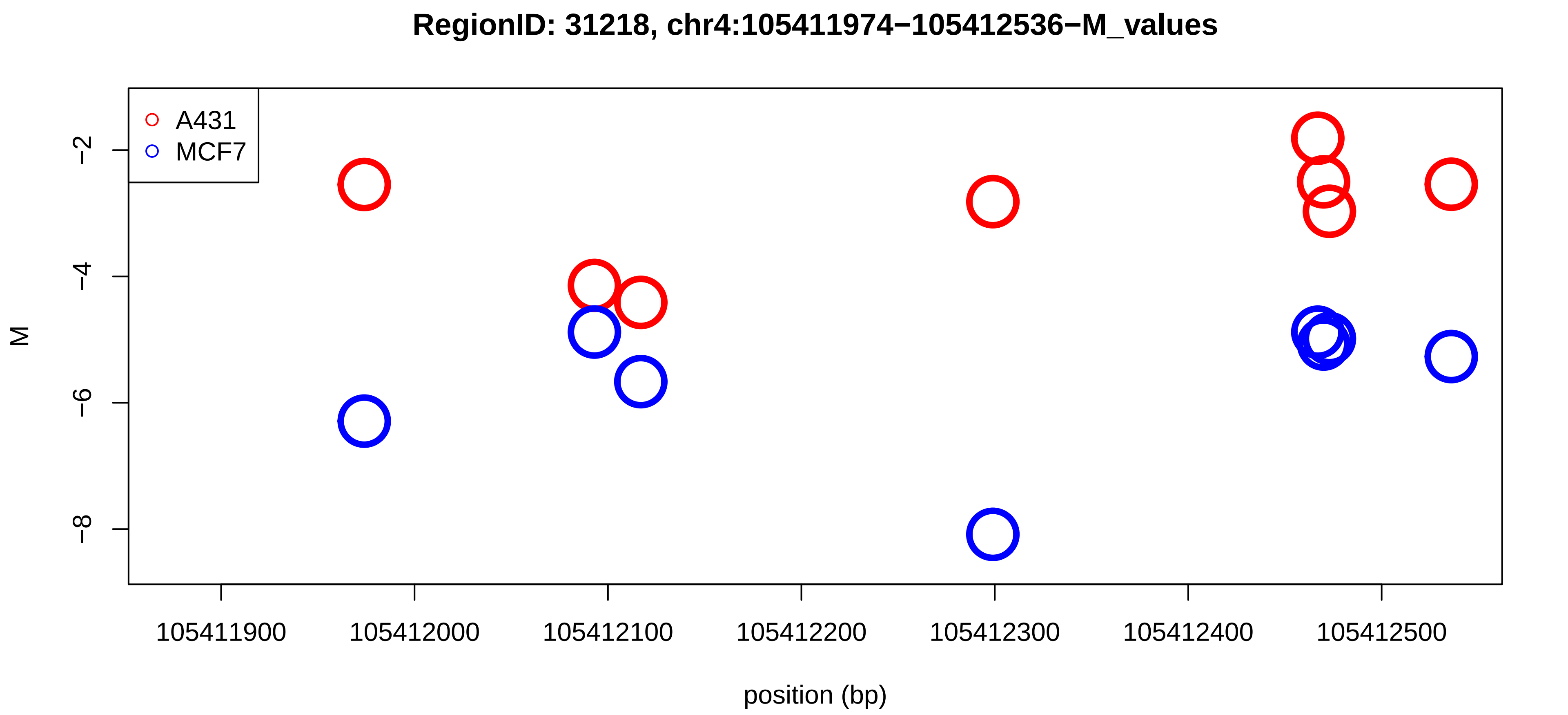

RegionID: 31218, chr4:105411974−105412536−M_values
A431
MCF7
−2
−4
−6
−8
M
105411900
105412000
105412100
105412200
105412300
105412400
105412500
position (bp)

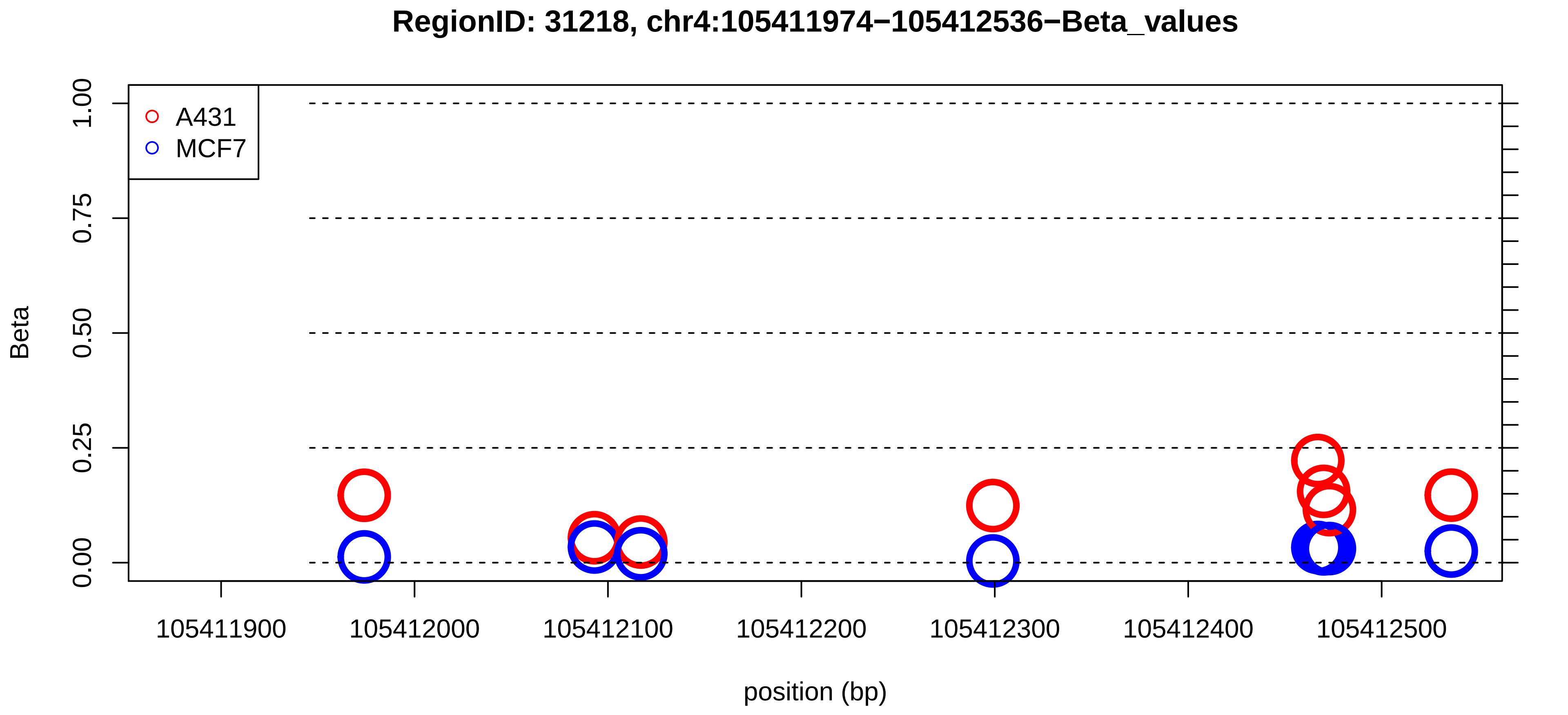

RegionID: 31218, chr4:105411974−105412536−Beta_values
A431
MCF7
1.00
0.75
0.50
0.25
0.00
Beta
105411900
105412000
105412100
105412200
105412300
105412400
105412500
position (bp)

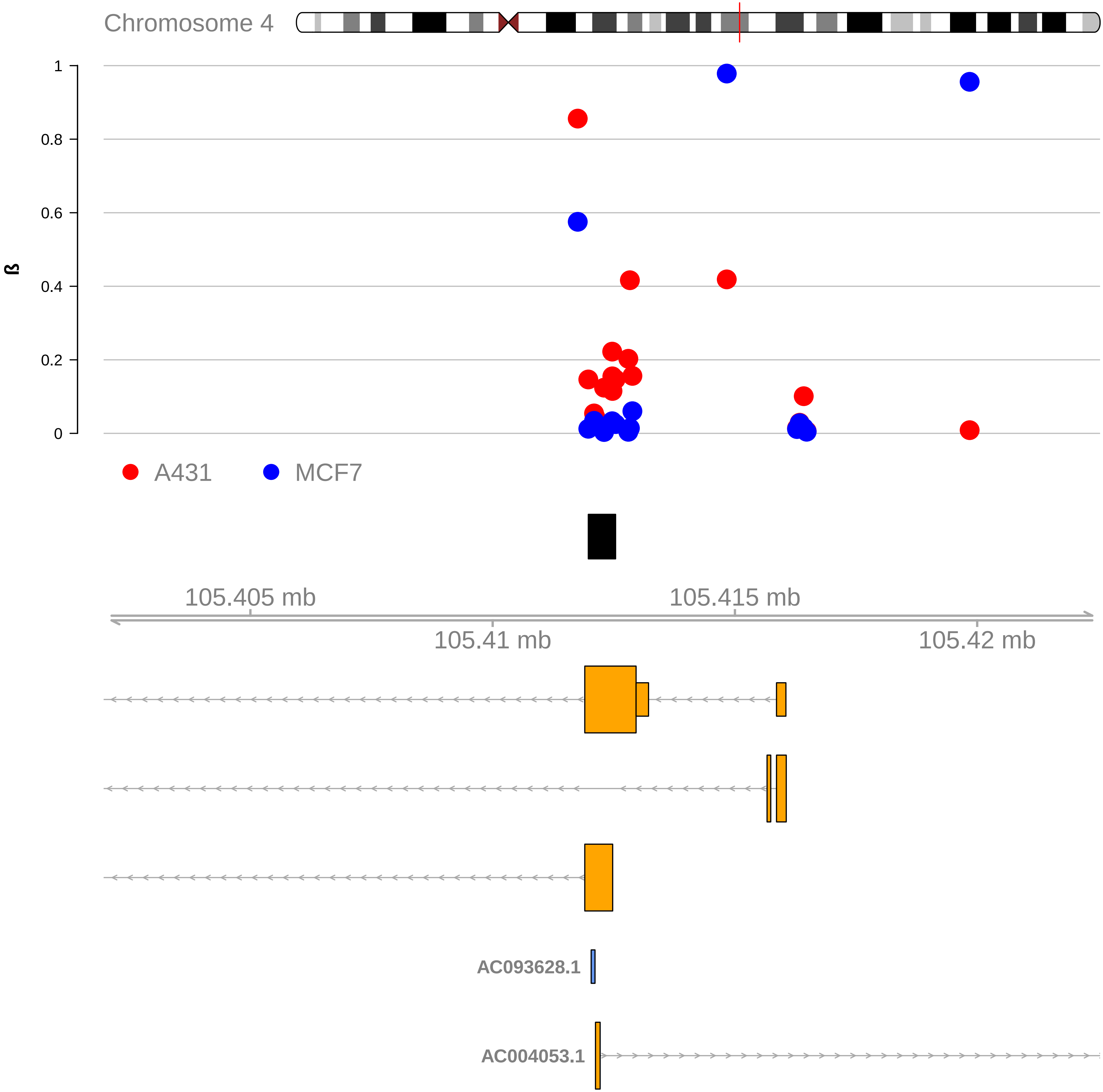

Chromosome 4
1
0.8
0.6
0.4
0.2
0
ß
A431
MCF7
105.405 mb
105.415 mb
105.41 mb
105.42 mb
AC093628.1
AC004053.1
